# Psychological well-being as part of the public health debate? Insight into dimensions, interventions, and policy

**DOI:** 10.1186/s12889-019-8029-x

**Published:** 2019-12-19

**Authors:** Claudia Trudel-Fitzgerald, Rachel A. Millstein, Christiana von Hippel, Chanelle J. Howe, Linda Powers Tomasso, Gregory R. Wagner, Tyler J. VanderWeele

**Affiliations:** 1 Department of Social and Behavioral Sciences, Harvard T.H. Chan School of Public Health, Boston, MA USA; 2 Lee Kum Sheung Center for Health and Happiness, Harvard T.H, Chan School of Public Health, Boston, MA USA; 3 Department of Psychiatry, Massachusetts General Hospital, Boston, MA USA; 4 Department of Psychiatry, Harvard Medical School, Boston, MA USA; 5 Wallace Center for Maternal, Child, and Adolescent Health, Berkeley School of Public Health, University of California, Berkeley, California USA; 6 Department of Epidemiology, Centers for Epidemiology and Environmental Health, Brown University School of Public Health, Providence, RI USA; 7 Department of Environmental Health, Harvard T.H. Chan School of Public Health, Boston, MA USA; 8 Department of Epidemiology, Harvard T.H. Chan School of Public Health, Boston, MA USA; 9 Department of Biostatistics, Harvard T.H. Chan School of Public Health, Boston, MA USA; 10 Human Flourishing Program, Harvard University, Boston, MA USA

**Keywords:** Purpose in life, Optimism, Mastery, Life satisfaction, Positive affect, Health, Mortality, Interventions, Randomized-controlled trials, Policy

## Abstract

**Background:**

Increasing evidence suggests that psychological well-being (PWB) is associated with lower disease and mortality risk, and may be enhanced with relatively low-cost interventions. Yet, dissemination of these interventions remains limited, in part because insufficient attention has been paid to distinct PWB dimensions, which may impact physical health outcomes differently.

**Methods:**

This essay first reviews the empirical evidence regarding differential relationships between all-cause mortality and multiple dimensions of PWB (e.g., life purpose, mastery, positive affect, life satisfaction, optimism). Then, individual-level positive psychology interventions aimed at increasing PWB and tested in randomized-controlled trials are reviewed as these allow for easy implementation and potentially broad outreach to improve population well-being, in concert with efforts targeting other established social determinants of health.

**Results:**

Several PWB dimensions relate to mortality, with varying strength of evidence. Many of positive psychology trials indicate small-to-moderate improvements in PWB; rigorous institution-level interventions are comparatively few, but preliminary results suggest benefits as well. Examples of existing health policies geared towards the improvement of population well-being are also presented. Future avenues of well-being epidemiological and intervention research, as well as policy implications, are discussed.

**Conclusions:**

Although research in the fields of behavioral and psychosomatic medicine, as well as health psychology have substantially contributed to the science of PWB, this body of work has been somewhat overlooked by the public health community. Yet, the growing interest in documenting well-being, in addition to examining its determinants and consequences at a population level may provoke a shift in perspective. To cultivate optimal well-being—mental, physical, social, and spiritual—consideration of a broader set of well-being measures, rigorous studies, and interventions that can be disseminated is critically needed.

## Background

Over the past decades, evidence for the mental and physical health benefits of enhanced psychological well-being (PWB) has expanded dramatically [1–3]. Notably, research in the fields of behavioral and psychosomatic medicine, as well as health psychology have substantially contributed to this body of work [1–7]. Yet, such work is still overlooked by a considerable proportion of the public health community, despite accumulating compelling reasons for a paradigm shift [8]. Associations of PWB levels with subsequent physical health outcomes have been well-documented [1, 2]. Easy-to-implement well-being interventions have been developed and evaluated in randomized-controlled trials (RCT), with many showing positive results [9, 10]. The potential for developing scalable interventions to be broadly disseminated is substantial and, in many cases, could require only limited or no professional training resources [9, 10]. Such interventions would improve not only PWB, but may have the potential to promote and maintain physical health as well [11]. This could be done in concert with efforts targeting other established social determinants of physical health/mortality (e.g., poverty, education, discrimination, social capital) [12, 13]. Existing skepticism among scientists may be due to insufficient attention paid to distinct dimensions of PWB (e.g., positive affect, optimism), which could differentially impact physical health and explain certain conflicting findings [2]. If PWB’s importance is to be embraced by the public health community and incorporated into policies, these distinctions need to be made clear. In this debate article, we argue that PWB dimensions, including life purpose, personal growth, mastery, autonomy, *ikigai,* life satisfaction, positive affect, sense of coherence, and optimism, may relate differently to all-cause mortality, based on existing empirical evidence. We also discuss some available interventions promoting PWB and how these might be used and disseminated more broadly.

PWB is important not only because of its potential effects on physical health but also as its own end. The World Health Organization (WHO) defines *health* as a “state of complete physical, mental, and social well-being and not merely the absence of disease or infirmity.” Importantly, PWB reflects more than the mere absence of psychological distress, such as anxiety or depressive symptoms. Although there is an inverse correlation between self-reported positive and negative psychological states, most coefficients vary from small-to-moderate, but are generally not strong in magnitude [14–16]. Psychological distress and well-being also have distinct biological correlates, further supporting the idea that they are separate rather than mirrored constructs [15, 17–19]. Accordingly, a successful psychotherapeutic or pharmacological treatment of anxiety symptoms will decrease symptoms of psychological distress but will not *necessarily* translate into a greater sense of purpose in life, autonomy, or optimism.

If we are to take seriously the WHO’s integrative conceptualization of health, PWB should be embraced as a fundamental public health goal [8]. The adoption of policies and programs supporting PWB in individuals and groups requires an understanding of the potential community benefits of such efforts. Through our assessment of the research investigating the relationship between PWB and all-cause mortality, interventions to change PWB, and policy implications of well-being research, we aim to contribute to this understanding.

### Defining well-being

Well-being is a complex and multifactorial construct. Measures of well-being are sometimes divided into objective measures, which mostly refer to “standard of living,” and subjective measures, which capture psychological, social, and spiritual aspects and are based on cognitive and affective judgements individuals make about their lives [20]. When these measures concern psychological aspects (e.g., happiness), they are often referred to as measures of psychological well-being (PWB). While certain PWB dimensions such as life satisfaction are often imbedded in “quality of life” measures, this latter multidimensional construct is much broader and includes other aspects related to mental and physical health like perceived stress, functioning/disability status, and physical symptoms [21, 22]. PWB on its own has been a central area of research in psychology for decades [7]. It is also important to epidemiology, to understand its contribution to health outcomes [8], and more broadly to public health, notably to implement country-level monitoring and policies promoting overall health [7].

Distinct theoretical dimensions have been proposed to characterize PWB research thus far (see [1] for details) including: hedonic well-being (e.g., feeling happy), evaluative well-being (e.g. being satisfied with life), eudaimonic well-being (e.g., finding purpose in life, having a sense of mastery and autonomy in one’s own decisions), and other constructs that contribute to feeling whole or well (e.g., optimism). In the following section, we describe these PWB dimensions and illustrate their potential effects on physical health by examining the evidence on how each is associated with all-cause mortality. Even though composite PWB measures exist, some authors have shown that it remains difficult to measure PWB across a continuum (unidimensionally) [23] and others have insisted on understanding PWB as a multidimensional, rather than unidimensional, construct [24]. Moreover, research has documented statistically significant associations among PWB dimensions themselves, with magnitude of estimates varying from small to moderate when evaluated among adults from various countries [14, 25–28]. Overall, these findings suggest that although PWB dimensions may share a latent factor, they do represent distinct constructs.

## Psychological well-being and mortality

A growing body of evidence suggests that various PWB dimensions are associated with subsequent chronic diseases and mortality, and potential mechanisms explaining associations, including stress-buffering effects [1, 2] and healthier behaviors [1, 2, 5, 29]. For instance, prior epidemiological research has shown that individuals experiencing higher levels of optimism were more likely to subsequently engage in favorable habits (e.g., physical activity), reduce/cease detrimental ones (e.g., smoking), leading to an overall healthy lifestyle [30–33]; in turn, the adoption of such healthy habits may lower one’s risk of chronic diseases and mortality [1, 2]. However, it is not always clear whether these longitudinal relationships remain after rigorous confounder control, whereby a third factor, such as socioeconomic status (e.g., education, personal income), influences both PWB and health. Likewise, whether these longitudinal associations do not simply capture reverse causation, whereby health status drives PWB levels, is sometimes uncertain. However, considering premature mortality risk, an objective endpoint, offers some methodological strengths such as virtually no misclassification and research based on longitudinal design by nature of the outcome. Recent meta-analyses have suggested that life satisfaction, positive affect, meaning/purpose in life, and optimism are protective against premature mortality [34–36], though the quality of statistical adjustment for potential confounders in these studies was variable. Here, we briefly discuss evidence as to whether and how various PWB dimensions are prospectively associated with premature all-cause mortality, specifically. Searches of literature written in English or French within PubMed and PsycInfo databases targeted individual prospective and longitudinal studies evaluating the role of at least one PWB dimension with mortality risk. Additional studies were obtained through bibliographies of eligible articles. Rigorous individual studies included in this narrative review all adjusted for baseline sociodemographics (e.g., age, sex, education), medical status (e.g., blood pressure, body mass index, chronic conditions), and health behaviors (e.g., smoking, physical activity). Some studies further adjusted for psychological distress, to determine PWB’s role on mortality beyond anxiety and depression symptoms, and for self-rated health.

### Purpose in life

Experiencing a sense of purpose and direction in one’s life has been consistently associated with reduced mortality. For instance, among 1236 older U.S. adults (mean age = 78 years), every standard deviation (SD) increase in life purpose was associated with 40% decreased hazard of 5-year mortality (hazard ratio, HR = 0.60; 95% confidence interval, CI = 0.42–0.87) [37]. In the Women’s Health Initiative cohort, after additional statistical control for psychological distress in multivariable models, greater life purpose was associated with lower likelihood of death over a 2-year period in 7675 older U.S. women [38]. Meta-analyses suggested similar effects (higher versus lower purpose in life; rate ratio, RR = 0.83, CI = 0.75–0.91) [35]. Some research has examined the role of *meaning* in life but the results are less convincing than those assessing purpose. A study of 1361 older U.S. adults (mean age = 79 years) over 5 years found no relationship of meaning in life with overall mortality (OR = 0.97; CI = 0.93–1.01) in multivariable models further adjusting for self-rated health [39]. These results raise the question of whether “meaning” and “purpose,” often used interchangeably, might capture distinct constructs that relate differently to mortality [40].

### Personal growth

To our knowledge, personal growth –that is whether individuals seek to realize their full potential and recognize that the self is constantly developing– has been explored in relation to mortality in only a handful of studies. Notably, in the Women’s Health Initiative investigation described above, personal growth levels were associated with lower 2-year mortality rates, both continuously (per 1-unit increase: HR = 0.95; CI = 0.93–0.98) and categorically (lower versus higher [reference group] quartile: OR = 2.10, CI = 1.42–3.08) [38]. This study also evaluated life purpose, with contrasting multivariable-adjusted estimates suggesting stronger associations with life purpose than personal growth (ORs = 3.55 versus 2.10) on mortality.

### Mastery

Mastery –whether individuals effectively manage their environments or perceive life as being under their control– has also been well-studied in relation to mortality. An investigation following 2829 Dutch adults (ages 55–85) for up to 3 years found that a 1-unit rise was associated with lower mortality odds (OR = 0.94, CI = 0.89–0.99), even after extensive adjustment of covariates including self-rated health, social support, self-efficacy, and self-esteem [41]. Likewise, among English adults from the EPIC-Norfolk Study (*N* = 20,495; ages 41–80), every 1-SD increase in mastery was associated with a lower rate of death (RR = 0.82, CI = 0.76–0.89) over 5 years, further controlling for psychological distress [42]. Similar results were obtained in U.S. samples too [43].

### Autonomy

Although research is sparse, available evidence suggests mortality risk is not strongly associated with autonomy, characterized as the extent to which individuals act independently without concern for external pressures. In a study of 9420 midlife British adults (mean age = 58 years) over a 5-year period, autonomy scores were unrelated to the hazard of death in multivariable models also controlling for self-rated health and psychological distress (per 1-unit increase: HR = 1.02; CI = 0.96–1.09) [44].

### Ikigai

This Japanese term translates into the happiness, worth, and benefit of being alive. It captures not only eudaimonic well-being (e.g., life purpose) but also hedonic well-being (e.g., pleasure), though usually assessed with only one item. Using data from the nationwide Japan Collaborative Cohort Study for Evaluation of Cancer Risk (*N* = 73,272; ages 40–79), adults with higher (versus lower) levels of *ikigai* had a reduced hazard of mortality over 5 years (HR_men_ = 0.80; CI = 0.72–0.89; HR_women_ = 0.80; CI = 0.69–0.92) [45]. In another Japanese cohort (*N* = 43,391; ages 40–79), lower and moderate *ikigai* levels (versus higher) were related to an increased 7-year hazard of death (HR_moderate_ = 1.1; CI = 1.0–1.2; HR_lower_ = 1.5; CI = 1.3–1.7), with further adjustment for self-rated health not altering these results [46].

### Positive affect

Feeling happy, joyful, cheerful, excited and proud are often included in the construct of positive affect. Data from the German Aging Survey (*N* = 3124; ages 40–85) showed that every unit increase in positive affect was associated with a lower 14-year mortality risk, after adjusting for sociodemographics, medical status, psychological distress, and also life satisfaction (HR = 0.81, CI = 0.70–0.93), though further controlling for self-rated health and physical activity attenuated the association (HR = 0.88, CI = 0.76–1.02) [47]. Even if happiness is a pleasurable feeling that is sometimes included in positive affect, it has also been studied on its own in prior PWB-mortality research. In a subset of the Million Women Study (*N* = 719,617; ages 53–72), English women who said they were “unhappy” or “usually happy” on a 1-item measure did not differ in mortality risk in 10-year follow-up compared to those who said they were “happy most of the time” (RR = 0.98, CI = 0.94–1.01; RR = 0.99, CI = 0.96–1.01, respectively) [48]. While this study has drawn media attention because of its large sample size and control for multiple covariates, its conclusions based on the use of a single happiness item have also has generated some controversy. Likewise, another study of older adults found no association between happiness assessed with 2 items and mortality [49]. These results may suggest that the comprehensive experience of various types of positive affect, rather than the sole experience of feeling happy as captured by single items, is what matters in terms of longevity.

### Life satisfaction

Life satisfaction can be measured either globally, capturing the extent to which individuals judge their life as a whole to be satisfactory, or specifically by life domains (e.g., work, family). A Canadian population-based study (*N* = 73,904; ages 18 to > 80) revealed that “very dissatisfied” (versus “very satisfied or satisfied”) individuals had an increased mortality risk (HR = 1.70, CI = 1.16–2.51), after controlling for numerous relevant covariates [50]. In the German Aging Survey described above, mortality risk was reduced for each unit increase in life satisfaction after adjusting for sociodemographics, medical status, psychological distress and also positive affect (HR = 0.89, CI = 0.79–1.00), but became unrelated after additional controlling for self-rated health and physical activity [47]. Although the estimate appears stronger with positive affect than life satisfaction in this study, even after including both in statistical models, these dimensions were assessed with distinct scales and scores were not standardized, which precludes formal comparison.

### Sense of coherence

One of the most rigorous early studies evaluating sense of coherence’s role in mortality risk has been conducted in the EPIC-Norfolk Study data (*N* = 16,668; ages 41–80) [51]. Sense of coherence was captured by the sum of 3 items measuring, respectively, the level of manageability, comprehensibility, and meaningfulness in one’s life. Adults with higher (versus lower) sense of coherence had a reduced risk of 6-year mortality (RR = 0.76, CI = 0.64–0.90), after statistical control for multiple covariates including psychological distress. These results have been replicated in a recent study of 585 men who were followed for 22 years and completed a more comprehensive assessment of the three constructs above [52]. Yet, it remains unclear whether any protective effects on mortality risk should instead be attributed to other PWB constructs captured by this scale. Notably, the meaningfulness item (“*Do you usually feel that your daily life is a source of personal satisfaction*?”) might relate to lower mortality risk because it captures, in fact, life satisfaction.

### Optimism

Multiple investigations indicate that dispositional optimism—a person’s general expectation that the future will turn out well or that good things will happen in the future—is associated with lower mortality rates. The Women’s Health Initiative (*N* = 97,253; ages 50–79) showed that higher versus lower quartiles of optimism were related to a reduced hazard of mortality over 8 years (HR = 0.86, CI = 0.79–0.93), after adding psychological distress to multivariable models [53]. Analyses conducted in another cohort of midlife U.S. women, the Nurses’ Health Study, replicated these results using the same research design [54]. Additionally, a Netherlands-based study among men and women ages 65–85 (*N* = 941) found a similar pattern over a 9-year period (HR_higher versus lower quartiles_ = 0.71; CI = 0.52–0.97), although results were not adjusted for psychological distress [55]. Altogether, these estimates are comparable to those reported by a recent meta-analysis (higher versus lower optimism; RR = 0.86; 95% CI, 0.80–0.92) [36].

### Overall psychological well-being

Other authors have considered global measures of psychological well-being. For instance, in a subset of the Midlife in the United States Study (*N* = 3032; ages 25–74), scores on items assessing positive affect, life satisfaction, eudaimonic well-being and social well-being were combined to capture positive mental health—also labeled *flourishing* by the authors [56]. Multivariable findings indicated that lower versus higher flourishing levels were related to greater odds of 10-year mortality (OR = 1.62; CI = 1.00–2.62). While combining various components of PWB may form a stronger predictor of subsequent health [57], these composite scores also somewhat limit our understanding of the specific dimensions that matter and the recommendations for future interventions.

### Summary

Overall, existing literature indicates that several PWB dimensions are associated with a reduced risk of premature all-cause mortality among the general population, with small to medium effects. These relationships were observed in studies with large sample sizes and over short to long follow-up periods. Associations were robust to adjustment for numerous covariates, including potential mechanisms that could explain associations (e.g., health behaviors); for some dimensions, associations were obtained despite the use of distinct PWB measures (e.g., optimism, sense of coherence). Among the dimensions reviewed, purpose in life, optimism, and *ikigai*, had the strongest evidence, followed by life satisfaction, positive affect, mastery, and sense of coherence. Available results with happiness, personal growth, and autonomy suggested no effect or were too limited to draw firm conclusions. Other PWB dimensions, including self-acceptance and emotional vitality, may have been investigated with all-cause mortality risk using prospective research designs, but studies using rigorous control for traditional medical and behavioral risk factors are scarce.

All studies reported above carefully controlled for sociodemographics, medical status, and health behaviors, and even after further adjustment for psychological distress, associations were generally evident, which further supports PWB as distinct from the absence of psychological distress. When more than one PWB dimension was investigated, however, very few authors evaluated their independent roles by including dimensions simultaneously in the models [47]. Thus, while these PWB factors appear conceptually distinct, it remains uncertain whether they independently reduce all-cause mortality and if so, the relative magnitude of their effects. When adjusting for self-rated health, some of the studies of certain domains, though not all, indicated null estimates. Self-rated health usually assesses, via one item, whether individuals perceive their health as excellent, very good, good, fair or poor, and is one of the strongest predictors of future morbidity and mortality risk [58]. However, controlling for self-rated health may sometimes be an overadjustment, because this rating is both defined and influenced by functional health, physical conditions, and most importantly, psychological distress and well-being [58]. Nevertheless, those PWB dimensions that are associated with lower mortality even after adjustment for self-rated health arguably manifest even stronger evidence for a causal relationship.

#### Psychological well-being and other outcomes

Although our narrative review focused on mortality, it is worth briefly noting that PWB may have important effects on numerous other outcomes. Observational and experimental research indicates that greater PWB levels are related to lower risk of cardiometabolic diseases, infectious illness and physical decline, though results with cancer are less clear [1, 2, 54]; PWB has also been related to more favorable health behaviors and healthier biological processes, which could act as mechanistic pathways relating PWB to chronic disease and mortality risk [1, 2, 29, 33]. Observational and experimental research also suggests PWB relates to higher future levels of employment, income, and work retention, as well as greater social support later on [59]. Likewise, prospective observational studies show that low PWB levels, including dimensions like self-acceptance, autonomy, life purpose, positive relationships, and mastery, are associated with greater likelihood of clinical depression 10 years later, after controlling for baseline traditional risk factors and psychological distress [60]. PWB was predictive of post-treatment symptom severity and remission status, independent of initial symptoms of depression and anxiety, in a recent clinical trial evaluating the effectiveness of cognitive-behavioral therapy for anxiety disorders [61]. PWB is not simply the absence of mental illness, and, in fact, contributes to subsequently preventing its onset and relapse. Moreover, PWB is desired not primarily because of its effects on mental and physical health, but as an end itself [57]. Most people want to be happy, satisfied with their life, and pursue a life that has meaning. PWB is thus important in its own right.

## Interventions

Albeit approximately 30% of one’s PWB is explained by heritable/dispositional factors, it is clear that external life events and environmental influences can account for a large proportion of an individual’s PWB. For instance, it has been well documented that greater levels of PWB are associated with higher levels of education, income, occupational status, and social capital [3, 7, 62, 63]. Intentional choices and behaviors, such as self-regulation and lifestyle habits, are also important determinants of PWB [5, 63]. Positive psychology (PP) thus appears as a compelling intervention strategy, as it aims to improve the frequency and intensity of positive emotional experiences, including optimism, gratitude, purpose/satisfaction in life, and positive affect, through intentional actions in the form of targeted, structured activities [9, 64, 65]. While these interventions aim to improve PWB within individuals, individuals are not the sole responsible agent of such changes; in fact, leveraging community and institutional resources is also increasingly encouraged to promote all individuals’ PWB by making strategies accessible to diverse groups of the population. In this regard, various PP interventions have been evaluated and have shown to improve mood and well-being among different populations [7, 9, 10, 65].

At the individual level, PP interventions are typically assigned, either separately or in combination, on a short-term regular basis (e.g., weekly) for participants to complete on their own, and then, in some cases, reviewed with a clinical or research professional to further elicit PWB [66]. Individual, group, and self-help interventions, including acts of kindness, counting blessings, and mindfulness, were first evaluated in non-clinical samples (e.g., community, students; examples in Table 1 with complete references in the Additional file 1) [9, 63, 64].

**Table 1:** Individual-based PP interventions evaluated in randomized-controlled trials

Positive Psychology (PP) intervention	Psychological well-being and other psychosocial outcomes effectively changed	Example references
Gratitude (e.g., gratitude for positive events, counting blessings, gratitude visit, or gratitude letter)	Life satisfaction (↑), optimism (↑), positive affect (↑), happiness (↑), depression symptoms (↓), negative affect (↓)	A1- A5
Best possible self	Life satisfaction (↑), optimism (↑), positive affect (↑)	A4, A6-A8
Acts of kindness	Life satisfaction (↑), optimism (↑), anxiety symptoms (↓), social connection (↑)	A5, A9
Use of character strengths	Happiness (↑), depressive symptoms (↓)	A3
Savoring or capitalizing on positive events	Positive affect (↑), life satisfaction (↑), depression symptoms (↓)	A10
Forgiveness	Positive affect (↑), anxiety symptoms (↓), depression symptoms (↓)	A11, A12
Mindfulness	Positive affect (↑), post-traumatic growth (↑), perceived stress (↓), depression symptoms (↓), anxiety symptoms (↓), quality of life (↑)	A13-A16
Multicomponent PP interventions (i.e., two or more interventions as those reported above, provided across several sessions)	Positive affect (↑), depression symptoms (↓), anxiety symptoms (↓), quality of life (↑)	A17-A23

*Note.* ↑= increase; ↓=decrease

In an early meta-analysis of 49 randomized or quasi-experimental studies (*N* = 4235), such PP interventions improved well-being, with a small but clinically meaningful mean effect size (*r* = 0.29, CI = 0.21–0.37) [64]. A more recent meta-analysis of 39 RCTs (*N* = 6139) [9] showed a similar effect of PP interventions on PWB (Cohen’s *d* = 0.20, CI = 0.09–0.30), with strongest effects for strategies targeting optimism, gratitude, and kindness [67], and with gains persisting for up to 6 months post-intervention (*d =* 0.16, CI = 0.02–0.30). Comparable effects are observed among clinical populations. A meta-analysis of 30 studies (*N* = 1864) in participants with either a psychiatric disorder (e.g., depression, anxiety) or a somatic condition (e.g., cardiometabolic disease, cancer) indicated that PP interventions had a small but meaningful effect on PWB (Hedges’ *g* = 0.24, CI = 0.13–0.35) [65]. Yet, it is still unclear whether longer-term health outcomes, including disease incidence and premature mortality, may be altered by improving PWB through these brief PP interventions, or if longer, more intensive interventions would be required [2].

Considering PP interventions at the institutional level is also critical. Because even changes of small magnitude at the individual level may translate into large changes at the population level, the potential benefits of such interventions on mental and physical health, including mortality risk, may be substantial. For instance, recent research has estimated a 5% decreased risk of stroke for individuals endorsing higher vs. lower levels of optimism, via optimism’ role on healthy lifestyle [33]. Such reduction in risk would indeed have major repercussions on a population’s health and economy*.*

In 2018, a public health summit of experts in mental and occupational health urged for building scientific evidence in the workplace that supports specific interventions aiming to improve and maintain employees’ health, including PWB [68]. Practices supporting, for instance, work-life balance and a physically/psychologically safe environment contributed to job satisfaction, independently of wages [20, 68]. Because employees’ general sense of well-being, beyond job satisfaction, could contribute to productivity and profitability [20, 68], broadly defined well-being interventions are increasingly evaluated in organizational settings. While the number of workplace-related RCTs is comparatively fewer, preliminary results are encouraging. A recent systematic review of RCTs and quasi-experimental studies indicated that PP interventions in the workplace were the only brief interventions to have a meaningful, albeit small, impact on employees’ mental health and well-being, whereas no evidence was found for strategies like relaxation and massage [69]. A subsequent RCT tested a 5-week online PP intervention adapted for the workplace among U.K. government employees (Table 2) [10]. Participants receiving the intervention (*n* = 170; vs. wait-list control group, *n* = 160) reported enhanced levels of positive affect and flourishing (*p <* .05), but not life satisfaction, post-intervention [10], reinforcing further empirical attention to PWB facets separately.

**Table 2:** Institution-based PP interventions evaluated in randomized-controlled trials

Positive Psychology (PP) intervention	Psychological well-being and other psychosocial outcomes effectively changed	Example references
*Workplace*		
Goal setting and planning	Positive affect (↑), flourishing (↑)	A24
*School*		
Gratitude	Positive affect (↑)	A25
Acts of kindness	Positive affect (↑), engagement (↑)	A25
Multicomponent PP interventions (e.g., acts of kindness, gratitude for positive events, mindfulness; PP interventions are often combined with some strategies from cognitive-behavioral therapy or acceptance and commitment therapy)	Psychological well-being (↑), flourishing (↑), personal growth (↑), optimism (↑), anxiety symptoms (↓) , depression symptoms (↓)	A26-A28

*Note.* ↑= increase; ↓=decrease

Besides the workplace, institution-based RCTs have also been conducted in schools (examples in Table 2 with complete references in the Additional file 1). While most studies have evaluated multicomponent interventions, making it difficult to disentangle the contribution of specific strategies, beneficial effects on PWB and other psychosocial outcomes were often observed. Other interventions relying on cognitive-behavioral strategies, like the Penn Resiliency Program, have been successful in improving psychosocial outcomes, including PWB, in schools and other settings (e.g., U.S. Army, see details in [11]).

## Policy implications

Over the past decade, governments from a dozen countries have also initiated regular well-being surveys as a component of public health data collection. Some countries evaluate hedonic PWB through a four-to-six domain questionnaire. Notably, in Bhutan, PWB is evaluated every few years with items like “*All things considered, how satisfied are you with your life as a whole these days?*”, along with other complimentary domains including social support, negative emotional experience, and spirituality. Likewise, the U.K. national survey includes a similar life satisfaction question, as well as items probing meaningful activities and positive/negative affect. Other national surveys use broader, culturally-relevant indices or objective well-being measures that capture infrastructure and services, environment and landscape factors, social relationships and even trust in government (e.g., Italy, Israel, Canada). International well-being surveys sometimes issue an annual “happiest country on Earth.” This judgment pleases not only the popular press, but also national governments that increasingly recognize that well-being measures can be a crude but reliable indicator of overall citizen satisfaction. Results from these surveys, after being reported to national assemblies, may also subsequently spur policy interventions. For example, the U.K. initiated the 24-h, free and confidential helpline, “Silver Line”, in 2013 in response to survey feedback of decreasing social connectedness among the aged [70]. Over 5 years, 2 million calls were received and over 70% reported that the helpline not only enhanced their social lives but also their happiness [70]; the U.K.’s first Minister of Loneliness was subsequently appointed in 2018.

Besides the importance of systematic monitoring of well-being indicators at the population level, implementing effective well-being policies is key to having a broader outreach in addition to individually tailored interventions. Notably, the Health-in-All-Policies (HiAP) approach, originating in South Australia, Europe, and Canada, has introduced a strategic way to better tackle social determinants of health, as documented in the 2010 Adelaide Statement [71]. This administrative process, more recently adapted by U.S. state and local governments, integrates health as a central outcome of all departments regardless of their functional oversight. Consequently, all sectors (e.g., employment, parks and recreation, housing administrations) become responsible for health-related interventions (e.g., facilitating access to greenness), rather than relying solely on public health policies [72]. For instance, better transport opportunities (e.g., cycling and walking paths) and reducing environmental degradation (e.g., pollution) may be ensured by leveraging a collaborative workforce as well as cross-cutting information and evaluation systems [71]. Such collaborative approaches can in turn enhance a population’s physical health more efficiently, via downstream consequences on common risk factors (e.g., obesity) and chronic conditions (e.g., cardiovascular diseases). HiAP could be improved by further integrating well-being science, including brief and relatively low-cost empirically-based PP interventions, into such municipal- and state-led strategies. Even though effects observed in individual-level RCTs are small in magnitude, such improvements in PWB could translate into notable changes at the population-level.

In parallel, policy strategies should address “the causes of the cause,” namely upstream social determinants that may drive PWB per se. As briefly mentioned previously, higher levels of education, income, occupational status, and social capital [3, 7, 62, 63], to name a few, have been associated with enhanced levels of PWB. Coordinated government actions, notably via the HiAP approach, tackle such social determinants. For example, working towards educational attainment and employment stability across various sociodemographic groups would not only create engaged citizens and promote better physical health, but also potentially increase their PWB [71]. Additionally, anti-discrimination policies, including the Equal Employment Opportunity Act, have historically helped to minimize group-based disparities in the social determinants of mental and physical health [73]. Therefore, stronger enforcement of anti-discrimination policies might be another way to alter downstream PWB. Efforts to support families and opportunities for community participation could likely increase levels of PWB as well [8, 57]. Lastly, because economic motives may act as a barrier to seeking mental health support, adequate reimbursement of psychotherapy services could also be implemented to enhance PWB [74].

Existing community initiatives might be disseminated across the country as well. Among others, the Office of Civic Wellbeing located in and supported by the city of Santa Monica, California, has launched the Wellbeing Project in 2013 [75]. This groundbreaking model for city governments uses the science of well-being to document community’s strengths and needs, along with the multiple determinants involved, to improve collective well-being. Moving from data to action, the Office has now various ongoing projects dealing with social determinants of PWB. One of them, in partnership with the Los Angeles County Department of Public Social Services, enrolls eligible Santa Monica residents for “CalFresh,” a public benefit program that supports individuals to meet their nutritional needs and improve healthy eating [75].

## Limitations and future avenues

PWB has promising potential to improve mental and physical health, derived from epidemiological studies and clinical trials described above. Although the current review was comprehensive but non-systematic by nature, some limitations were evident and should guide future research and practice. Firstly, PWB-mortality associations have been rarely investigated across sociodemographic groups (i.e., by explicitly evaluating effect modification, beyond statistical adjustment), and many interventions have been restricted to clinical or convenience samples, mostly in high-income countries, which may not be generalizable to other populations. Yet, preliminary observational findings from these studies hint at effect modification by sex [34, 45], race/ethnicity [53], educational attainment [43], as well as specific causes of death (e.g., cardiovascular versus cancer) [42, 45, 53]. As for age, insight about the role of PWB, as experienced during childhood or adolescence, in health would be informative from a lifecourse perspective. However, most epidemiological cohorts have not queried PWB indicators in early life, and studies in younger individuals do not have the required follow-up to evaluate PWB’s role in mortality.

With regard to lower-middle-income countries, a handful of studies have examined the interplay between mental and physical health. However, to our knowledge they either have not collected data on PWB indicators specifically to date (e.g., the Kenyan Grandparents Study) or did not yet investigate PWB’s role in mortality, most likely because they were initiated recently (e.g., the Brazilian Longitudinal Study of Aging, Health and Aging in Africa: A Longitudinal Study of an INDEPTH Community in South Africa). Besides country-level income, the role of other indicators of socioeconomic status (SES) in the PWB-mortality relationship specifically is less known. In fact, although most rigorous studies cited above have controlled for education level, fewer investigations have adjusted for individual/family income [38, 44, 53], occupation status/types [42, 45, 46, 51, 53, 55], or area deprivation [48], and did not formally assess effect modification. Hence, it remains unclear as of now whether findings obtained from studies assessing the PWB-mortality association in high-income countries and adjusting for certain SES indicators may generalize to those of lower-middle-income countries and other socioeconomic groups.

Furthermore, rigorous methodologies should be favored (e.g., lagged analyses to address potential for reverse causation, repeated PWB measurements to capture changes, comprehensive set of covariates to account for confounding, simultaneous adjustment for multiple dimensions of PWB). In addition to improving methodological rigor, systematically incorporating well-being scales in large national cohort studies will help solidify the evidence of PWB’s causal role in health outcomes [8].

Of course, PWB measure selection depends on the context. For instance, for a multi-purpose epidemiological cohort study, with limited space on the questionnaires, or for studies in which PWB is investigated only as an outcome, a composite PWB measure might be sufficient. However, to advance science and be more precise, and consistent with the argument that PWB is a multidimensional rather than unidimensional construct detailed above, dimension-specific measures should be favored. To date, numerous large-scale studies have administered at least one PWB measure to their participants (e.g., Women’s Health Initiative cohort, Nurses’ Health Study, Midlife in the United States Study, Health and Retirement Study, Longitudinal Aging Study Amsterdam, EPIC-Norfolk Study, Japan Collaborative Cohort Study). Including additional PWB measures in these studies, to permit comparison across constructs, and expanding PWB assessments to other large national cohorts is warranted. Consequently, such evidence will guide the development of more targeted and efficient intervention, as well as primary/primordial prevention strategies. For instance, PP interventions implemented earlier in the lifecourse may have the potential to reduce adverse behaviors and detrimental biological processes over time, possibly lowering likelihood of chronic illness later in life.

Lastly, additional research exploring whether and how well-being strategies and policies can be implemented in communities will be needed to achieve a population-level impact. Notably, health professionals should assess the barriers and benefits of integrating PWB into standard clinical practices focused on deficits and disorders. Leveraging input from local agents who grasp the needs and characteristics of certain subgroups would facilitate the crafting and delivery of empirically-based PP interventions (e.g., teachers in targeted schools of low-SES neighborhoods). Eventually, public health policy-makers will have to evaluate the cost-effectiveness of implementing PP interventions in these distinct environments (e.g., medical settings, schools, neighborhoods) [76].

## Conclusions

Existing research to date suggests that many, though not all, dimensions of psychological well-being (PWB) are associated with all-cause mortality. Building from the evidence of associations between PWB and mortality, this essay then discusses interventions to promote PWB. Many randomized-controlled trials evaluating positive psychology interventions at the individual level indicate small-to-moderate improvements in various PWB dimensions; rigorous institution-level interventions are comparatively few, but preliminary results suggest benefits as well. These interventions have the potential to be easily implemented and, in turn, have a broad outreach to improve population well-being. Existing health policies geared towards the improvement of population well-being could also leverage the science of PWB.

While this body of work has been overlooked by part of the public health community [8, 11], the growing interest in documenting well-being, in addition to examining its determinants and consequences at a population level may provoke a shift in perspective. Over the past decade, numerous countries have initiated well-being assessment via national surveys, which have led to the implementation of some institutional policies geared towards PWB’s enhancement. However, there is at present no attempt at national measurement in the U.S.; it is perhaps time that this be changed. To cultivate optimal well-being—mental, physical, social, and spiritual—consideration of a broader set of well-being measures, rigorous studies, as well as public and private interventions is critically needed.

## Supplementary information

**Additional file 1:** Online supplement – references of randomized-controlled trials

## Data Availability

Data sharing is not applicable to this article as no datasets were generated or analyzed during the current study.

## Abbreviations

CI: Confidence interval
HiAP: Health-in-All-Policies
HR: Hazard ratio
OR: Odd ratio
PP: Positive psychology
PWB: Psychological well-being
RCT: Randomized-controlled trial
RR: Relative risk
SD: Standard deviation
U.K: United Kingdom
U.S: United States
