# Supplementary material for: Psychological well-being as part of the public health debate? Insight into dimensions, interventions, and policy
Source: BMC Public Health. 2019 Dec 19;19:1712. doi: 10.1186/s12889-019-8029-x (PMC6923969; doi:10.1186/s12889-019-8029-x)
Supplement: Supplementary file 1 — Additional file 1: Online supplement – references of randomized-controlled trials [file 12889_2019_8029_MOESM1_ESM.docx]

**Additional file 1 – REFERENCES OF RANDOMIZED-CONTROLLED TRIALS**

1. Boehm JK, Lyubomirsky S, Sheldon KM. A longitudinal experimental study comparing the effectiveness of happiness-enhancing strategies in Anglo Americans and Asian Americans. *Cogn Emot.* 2011;25(7):1263-1272.

2. Emmons RA, McCullough ME. Counting blessings versus burdens: An experimental investigation of gratitude and subjective well-being in daily life. *Journal of personality and social psychology.* 2003;84(2):377-389.

3. Seligman ME, Steen TA, Park N, Peterson C. Positive psychology progress: Empirical validation of interventions. *The American psychologist.* 2005;60(5):410-421.

4. Sheldon KM, Lyubomirsky S. How to increase and sustain positive emotion: The effects of expressing gratitude and visualizing best possible selves. *The Journal of Positive Psychology.* 2006;1(2):73-82.

5. Kerr SL, O’Donovan A, Pepping CA. Can gratitude and kindness interventions enhance well-being in a clinical sample? *Journal of Happiness Studies* 2015;16:17-36.

6. King LA. The health benefits of writing about life goals. *Personality and Social Psychology Bulletin.* 2001;27:798-807.

7. Layous K, Nelson SK, Lyubomirsky S. What is the optimal way to deliver a positive activity intervention? The case of writing about one's best possible selves. *Journal of Happiness Studies.* 2013;14:635-654.

8. Peters ML, Meevissen YMC, M. HM. Specificity of the best possible self intervention for increasing optimism: Comparison with a gratitude intervention. *Terapia Psicológica.* 2013;31(1):93-100.

9. Buchanan KE, Bardi A. Acts of kindness and acts of novelty affect life satisfaction. *J Soc Psychol.* 2010;150(3):235-237.

10. Hurley DB, Kwon P. Results of a study to increase savoring the moment: Differential impact on positive and negative outcomes. *J Happiness Stud.* 2012;13(4):579-588.

11. Goldman DB, Wade NG. Comparison of forgiveness and anger-reduction group treatments: A randomized controlled trial. *Psychother Res.* 2012;22(5):604-620.

12. Luskin FM, Ginzburg K, Thoresen CE. The efficacy of forgiveness intervention in college age adults: Randomized controlled study. *Humboldt Journal of Social Relations.* 2005;29:163-183.

13. Carlson LE, Tamagawa R, Stephen J, Drysdale E, Zhong L, Speca M. Randomized-controlled trial of mindfulness-based cancer recovery versus supportive expressive group therapy among distressed breast cancer survivors (MINDSET): Long-term follow-up results. *Psycho-oncology.* 2016;25(7):750-759.

14. Geschwind N, Peeters F, Drukker M, van Os J, Wichers M. Mindfulness training increases momentary positive emotions and reward experience in adults vulnerable to depression: A randomized controlled trial. *Journal of consulting and clinical psychology.* 2011;79(5):618-628.

15. Hazlett-Stevens H, Oren Y. Effectiveness of mindfulness-based stress reduction bibliotherapy: A preliminary randomized controlled trial. *J Clin Psychol.* 2017;73(6):626-637.

16. Jain S, Shapiro SL, Swanick S, et al. A randomized controlled trial of mindfulness meditation versus relaxation training: Effects on distress, positive states of mind, rumination, and distraction. *Ann Behav Med.* 2007;33(1):11-21.

17. Celano CM, Albanese AM, Millstein RA, et al. Optimizing a positive psychology intervention to promote health behaviors after an acute coronary syndrome: The Positive Emotions After Acute Coronary Events III (PEACE-III) randomized factorial trial. *Psychosomatic medicine.* 2018;80(6):526-534.

18. Cohn MA, Pietrucha ME, Saslow LR, Hult JR, Moskowitz JT. An online positive affect skills intervention reduces depression in adults with type 2 diabetes. *J Posit Psychol.* 2014;9(6):523-534.

19. Gander F, Proyer RT, Ruch W, Wyss T. Strength-based positive interventions: Further evidence for their potential in enhancing well-being and alleviating depression. *J Happiness Stud.* 2013;14(4):1241–1259.

20. Moskowitz JT, Carrico AW, Duncan LG, et al. Randomized controlled trial of a positive affect intervention for people newly diagnosed with HIV. *Journal of consulting and clinical psychology.* 2017;85(5):409-423.

21. Peters ML, Smeets E, Feijge M, et al. Happy despite pain: A randomized controlled trial of an 8-week Internet-delivered positive psychology intervention for enhancing well-being in patients with chronic pain. *Clin J Pain.* 2017;33(11):962-975.

22. Schueller SM, Parks AC. Disseminating self-help: Positive psychology exercises in an online trial. *J Med Internet Res.* 2012;14(3):e63.

23. Huffman JC, Millstein RA, Mastromauro CA, et al. A positive psychology intervention for patients with an acute coronary syndrome: Treatment development and proof-of-concept trial. *J Happiness Stud.* 2016;17(5):1985-2006.

24. Oliver JJ, MacLeod AK. Working adults' well-being: An online self-help goal-based intervention. *J Occup Organ Psychol.* 2018;91(3):665-680.

25. Ouweneel E, Le Blanc PM, Schaufeli WB. On being grateful and kind: Results of two randomized controlled trials on study-related emotions and academic engagement. *J Psychol.* 2014;148(1):37-60.

26. Ruini C, Ottolini F, Tomba E, et al. School intervention for promoting psychological well-being in adolescence. *J Behav Ther Exp Psychiatry.* 2009;40(4):522-532.

27. Burckhardt R, Manicavasagar V, Batterham PJ, Hadzi-Pavlovic D. A randomized controlled trial of strong minds: A school-based mental health program combining acceptance and commitment therapy and positive psychology. *J Sch Psychol.* 2016;57:41-52.

28. Schonert-Reichl KA, Oberle E, Lawlor MS, et al. Enhancing cognitive and social-emotional development through a simple-to-administer mindfulness-based school program for elementary school children: A randomized controlled trial. *Dev Psychol.* 2015;51(1):52-66.
